# Association of serum lactate with outcome after out-of-hospital cardiac arrest treated with therapeutic hypothermia

**DOI:** 10.1371/journal.pone.0173239

**Published:** 2017-03-10

**Authors:** Jean-Christophe Orban, Michaël Novain, Florian Cattet, Rémi Plattier, Mohamed Nefzaoui, Hervé Hyvernat, Olivier Raguin, Michel Kaidomar, Sébastien Kerever, Carole Ichai

**Affiliations:** 1 Medical surgical ICU, Pasteur 2 Hospital, Nice University Hospital, 30 Voie Romaine, Nice, France; 2 Medical ICU, Archet Hospital, Nice University Hospital, 151 route de Saint-Antoine, Nice, France; 3 Intensive Care Unit, Antibes General Hospital, 107 avenue de Nice, Antibes, France; 4 Intensive Care Unit, Fréjus Saint-Raphaël General Hospital, 240 avenue de Saint-Lambert, Fréjus, France; 5 Departments of Anesthesiology and Critical Care, Lariboisière University Hospital, 2 rue Ambroise Paré, Paris, France; Azienda Ospedaliero Universitaria Careggi, ITALY

## Abstract

**Aims:**

Lactate reflects hypoxic insult in many conditions and is considered as a prognosis factor. But, after cardiac arrest, its interest is still debated. Our study aimed to assess the prognosis value of lactate in out-of-hospital cardiac arrest patients treated with therapeutic hypothermia.

**Methods:**

This retrospective observational study included out-of-hospital cardiac arrest patients treated with therapeutic hypothermia in four ICUs. Lactate levels were compared at different times during the first 24 hours according to outcome at ICU discharge and to the type of death (multiorgan or neurologic failure).

**Results:**

Two hundred and seventy-two patients were included, 89 good outcome and 183 poor outcome. In the latter group, 171 patients died, from multiorgan failure in 30% and neurologic failure in 70%. Lactate levels were higher in the poor compared to the good outcome patients at admission (5.4 (3.3–9.4) vs. 2.2 (1.5–3.6) mmol/L; p<0.01), 12 hours (2.5 (1.6–4.7) vs. 1.4 (1.0–2.2) mmol/L; p<0.01) and 24 hours (1.8 (1.1–2.8) vs. 1.3 (0.9–2.1) mmol/L; p<0.01). Patients succumbing from multiorgan failure exhibited higher lactate levels compared to those dying from neurologic failure at admission (7.9 (3.9–12.0) vs. 5.2 (3.3–8.8) mmol/L; p<0.01), H12 (4.9 (2.1–8.9) vs. 2.2 (1.4–3.4) mmol/L; p<0.01) and H24 (3.3 (1.8–5.5) vs. 1.4 (1.1–2.5) mmol/L; p<0.01). Initial lactate levels showed an increasing proportion of poor outcome from the first to fourth quartile.

**Conclusions:**

After out-of-hospital cardiac arrest treated with therapeutic hypothermia, lactate levels during the first 24 hours seem linked with ICU outcome. Patients dying from multiorgan failure exhibit higher initial lactate concentrations than patients succumbing from neurological failure.

## Introduction

Cardiac arrest represents a major public health issue in Western countries accounting for about 600,000 cases per year in Europe and North America [1,2]. It remains the main cause of death in industrialized countries [3] with a very low survival rate at hospital discharge. Among successfully resuscitated patients admitted to intensive care unit (ICU) after cardiac arrest, less than 30% will be discharged from hospital [4]. This early mortality after resuscitation is mostly caused by post-resuscitation circulatory failure (mainly due to systemic ischemia–reperfusion) and post-anoxic brain injury. However, the prognostic determination remains challenging. Currently, no single tool can provide neurologic prognostication after out-of-hospital cardiac arrest (OHCA), which should be based on multiple predictors (EEG, biomarkers, imaging) depending on locally available tests and expertise [5]. Moreover, prolonged observation and repeated assessments are frequently mandatory since results of initial assessment are inconclusive. Finally the use of therapeutic hypothermia and associated sedation interfere with prognostication tools, especially clinical examination. An early and accurate marker of prognosis would be useful to identify patients who will most benefit from intensive care.

Lactate is a physiological substrate produced by pyruvate reduction during glycolysis. During hypoxia, glycolysis leads to accumulation of pyruvate and subsequently of lactate. Thus hyperlactatemia represents a good marker of tissue hypoxia. Single assessment or lactate clearance (i.e. decrease) has been used as a prognostic factor in different settings such as emergency department, ICUs [6,7] or in the perioperative period. During cardiac arrest, lactate production and concentration increases with the duration of downtime [8]. Thus, it has been proposed as a tool to determine the neurological prognosis of patients after OHCA [9,10]. However, subsequent studies reported conflicting results and were difficult to compare due to various percentages of application of therapeutic hypothermia and different proportions of in-hospital and out-of-hospital cardiac arrest in the population [11–16]. We therefore performed a study evaluating the association of serum lactate concentrations with outcome in out-of-hospital cardiac arrest patients treated with therapeutic hypothermia.

## Materials and methods

### 1. Patients selection and study design

Out-of-hospital cardiac arrest patients aged 18–80 years admitted in one of the participating ICUs between January 2006 and April 2013, and treated with therapeutic hypothermia were retrospectively reviewed using diagnosis codes. We excluded cardiac arrest patients from traumatic, toxic and neurologic causes. Patients’ records were anonymized before analysis. Four ICUs were involved in this study: 3 mixed and 1 medical ICUs. The national committee on data management (Commission Nationale Informatique Liberté) approved this study (decision DE 2013–055) and our hospital ethic committee waived the need for patient's consent.

### 2. Patients’ management

Patients were treated according to the ILCOR recommendations **[**17,18**]**. Briefly after return of spontaneous circulation, patients were admitted to one of the four participating ICUs. Therapeutic hypothermia was initiated as soon as possible using external or internal methods. Duration of hypothermia and target temperature were set according to guidelines. All patients were sedated with an association of midazolam and fentanyl during hypothermia and paralyzed using continuous infusion of cisatracurium. All patients were intubated and mechanically ventilated aiming at a PaO_2_ between 75 and 100 mmHg and PaCO_2_ between 35 and 45 mmHg. A central venous catheter and an arterial line were inserted for monitoring respectively central venous and arterial blood pressure, and blood sampling. Therapeutic goals were a mean arterial pressure > 80 mmHg and urine output > 0.5 mL/kg/hr. If needed, patients were given fluid infusion or catecholamines (dobutamine, norepinephrine, epinephrine) according to haemodynamic monitoring data. Haemoglobin concentration was kept > 10 g/dl. Neurologic outcome was assessed using the Cerebral Performance Category (CPC) scale ranging from 1 to 5, at ICU discharge. CPC 1 and 2 were considered as good outcome and CPC 3 to 5 as poor outcome.

### 3. Study protocol

We extracted from medical records the pre-hospital clinical data (age, sex, initial rhythm as shockable or non-shockable, durations of no-flow and low-flow) and ICU clinical data (beta-adrenergic drug use, outcome assessed by CPC scale at ICU discharge and mode of death). The mode of death was dichotomized as multiorgan failure (MOF) following hemodynamic compromise or neurologic failure leading to withdrawal or withholding of care as previously reported [19]. Arterial lactate concentrations were collected on admission in ICU and at subsequent times when available (12 and 24 hours after admission). As lactate declines quickly after hemodynamic stabilisation, we measured the time between cardiac arrest and first lactate measurement.

### 4. Statistical analysis

The data are described as their frequencies and percentages for the categorical variables, and as their medians (25th–75th percentile range) for the quantitative variables. Categorical variables were compared using the Chi-square test or the Fisher exact test, as appropriate, and quantitative variables using Wilcoxon's ranked-sum test. Cubic splines followed by a log transformation allowed the determination of threshold values used for the estimation univariate odds ratios (OR) and 95% confidence intervals (95% CI). All tests were two-sided and statistical significance was set at the *p* = 0.05 level. Analyses were performed using R open source software 3.1.1 (available online at http://www.R-project.org).

## Results

Two hundred and seventy-two patients met the study inclusion criteria at the 4 participating ICUs. From this population, 89 (33%) patients presented a good outcome at ICU discharge (65 considered CPC 1 and 24 as CPC 2) whereas 183 (67%) patients were considered poor outcome (6 considered CPC 3, 6 as CPC 4 and 171 as CPC 5). In the latter group, patients died from MOF in 30% (n = 52) and neurologic failure in 70% (n = 119). Baseline characteristics of all patients and according to outcome are reported in Table 1. As reported in Table 1, patients with a good outcome were younger and more likely to be male. They also presented shorter durations of no-flow and low-flow and a higher percentage of shockable rhythm.

**Table 1:** Baseline characteristics of the population and according to outcome at ICU discharge.

	All patients (n = 272)	Good outcome patients (n = 89)	Poor outcome patients (n = 183)	*p* value
Age (years)	65 (53–75)	60 (49–70)	68 (58–76)	0.002
Sex ratio				0.001
• Male	192 (71%)	75 (84%)	4%)	
• Female	80 (29%)	14 (16%)	66 (36%)	
No-flow (min)	3 (0–10)	1 (0–5)	5 (0–10)	<0.001
Low-flow (min)	15 (10–25)	10 (5–15)	20 (15–30)	<0.001
Origin				<0.001
• Cardiac	167 (63%)	70 (80%)	54%)	
• Respiratory	100 (37%)	18 (20%)	82 (46%)	
Initial rhythm				<0.001
• Shockable	105 (44%)	57 (74%)	30%)	
• Non Shockable	131 (56%)	20 (26%)	111 (70%)	

Data are expressed as median and interquartile range. A *p* value<0.05 was considered significant.

**Fig 1:**
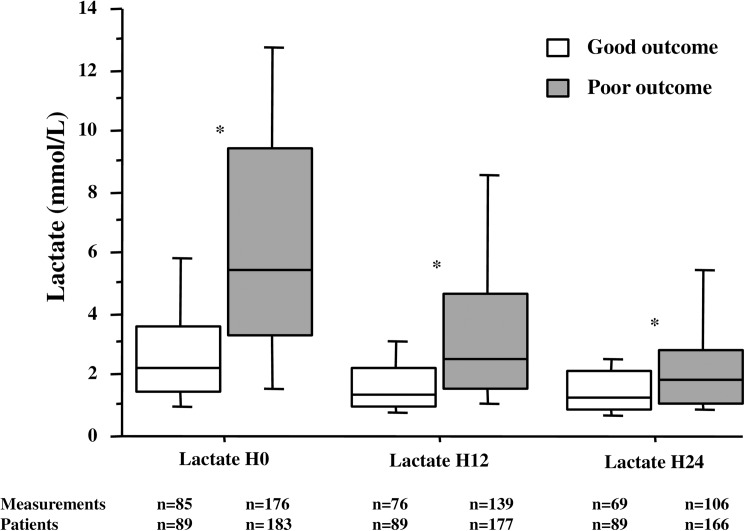
Arterial lactate levels during the 24-hours study period in good and poor outcomes groups. Data expressed as median and interquartile range. Comparisons between groups by Mann-Whitney test: * *p*<0.01.

**Fig 2:**
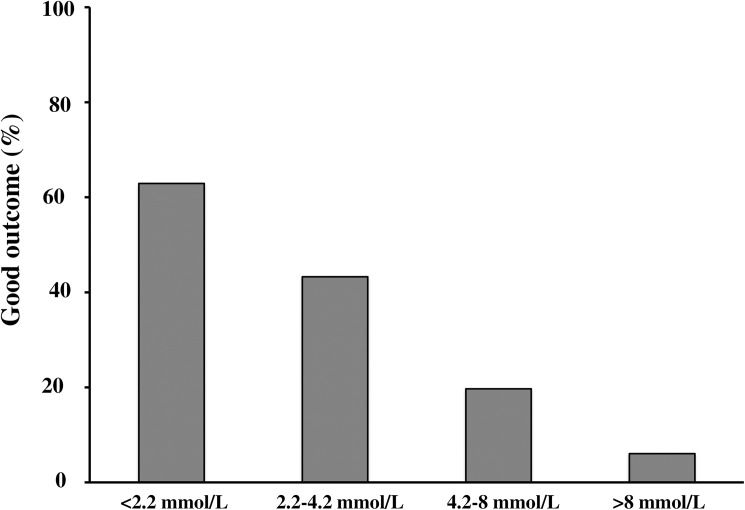
Quartiles of admission lactate levels and percentage of good outcome at ICU discharge. *p* value <0.001 for comparison between quartiles.

The time between cardiac arrest and first lactate measurement was 163 (120–221) minutes. This time was not different between the good and poor outcome populations (170 (120–240) vs. 160 (120–220) minutes; p = 0.89). Arterial lactate concentrations decreased in each group during the 24-h study period and were higher in the poor outcome compared to the good outcome patients at each time (Fig 1). To establish a relation between lactate levels and prognosis, the population was divided in quartiles according to initial values (Quartile 1: < 2.2 mmol/L; quartile 2: 2.2–4.2 mmol/L; quartile 3: 4.2–8 mmol/L; quartile 4: > 8 mmol/L). As depicted in Fig 2, the proportion of favorable outcome decreased gradually from the first to the fourth quartiles (p<0.01).

The main factors related to outcome are reported in the Table 2. The presence of a shockable rhythm and a cardiac origin seem associated to a better prognosis. On the contrary, a low-flow duration > 15 min, a lactate value at admission > 4 mmol/L and lactate value at H12 > 3 mmol/L show a strong association with a poor outcome. The sensitivities and specificities of the different lactate thresholds are respectively 0.66 and 0.80 at admission, 0.42 and 0.88 at 12 hours, and 0.33 and 0.88 at 24 hours. The area under the curve of lactate at the different time points are respectively 0.78 for admission, 0.72 for 12 hours and 0.64 for 24 hours.

**Table 2:** Main factors including lactate levels during the first 24 hours associated to poor outcome.

Parameters	Odds-ratio (95% CI)	*p* value
Age > 58 years	2.25 (1.33–3.81)	0.002
Female sex	3.02 (1.58–5.76)	0.0008
Cardiac origin	0.30 (0.17–0.55)	<0.0001
Respiratory origin	3.29 (1.81–5.96)	<0.0001
Shockable rhythm	0.15 (0.08–0.28)	<0.0001
No flow > 3 min	3.04 (1.63–5.64)	0.0004
Low flow > 15 min	5.27 (2.77–10.0)	<0.0001
Admission lactate > 4 mmol/L	7.54 (4.07–14.0)	<0.0001
H12 lactate > 3 mmol/L	5.73 (2.56–12.9)	<0.0001
H24 lactate > 2.5 mmol/L	3.54 (1.55–8.11)	0.003

Results are expressed as odds-ratio and 95% confidence intervals. A *p* value<0.05 was considered significant.

**Fig 3:**
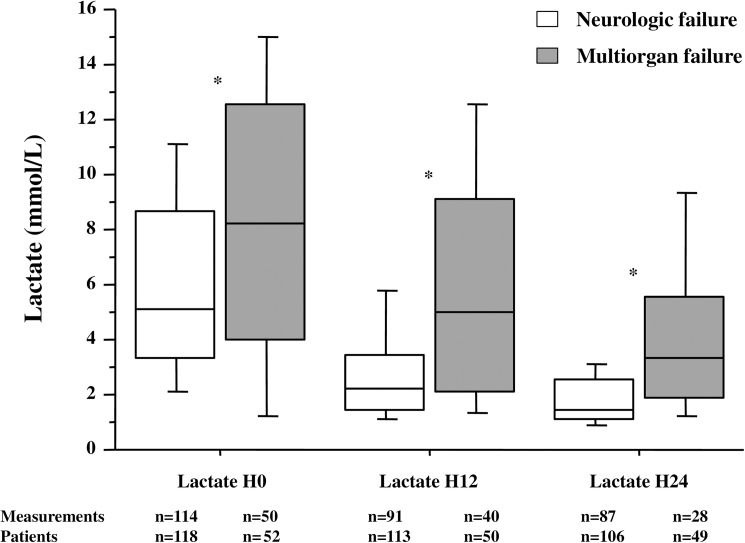
Arterial lactate levels during the 24-hours study period in MOF and neurologic failure groups. Data expressed as median and interquartile range. Comparisons between groups by Mann-Whitney test: * *p*<0.01

Arterial lactate levels from admission to 24 hours were compared between patients dying from MOF or neurologic failure. Arterial lactate concentrations decreased in each group during the 24-h study period and MOF patients exhibited higher arterial lactate levels at all times of the study compared to the neurologic failure patients (Fig 3).

The main factors related to the modes of death are reported in the Table 3. Interestingly, different parameters did not show the same association with MOF or neurologic failure. Indeed, a shockable rhythm seemed associated to a lower risk of death by neurologic failure. A norepinephrine rate > 0.4 g/kg/min was strongly associated to a death by MOF. Initial lactate > 4 mmol/L was associated to both modes of death whereas subsequent measurements were only significantly associated to death following MOF.

**Table 3:** Main factors associated to the type of death (MOF or neurological failure).

Parameter	Risk factors of death due to multiorgan failure (n = 52)		Risk factors of death due to neurologic failure (n = 119)	
	OR (95% CI)	*p* value	OR (95% CI)	*p* value
Shockable rhythm	0.63 (0.32–1.24)	0.18	0.25 (0.14–0.44)	<0.0001
No flow duration > 3 min	1.25 (0.59–2.68)	0.56	2.23 (1.24–3.99)	0.007
Low flow duration > 15 min	1.80 (0.96–3.37)	0.067	2.33 (1.39–3.92)	0.001
Norepinephrine > 0.4 μg/kg/min	3.39 (1.79–6.42)	0.0002	1.15 (0.69–1.90)	0.59
Admission lactate > 4 mmol/L	3.08 (1.57–6.04)	0.001	2.49 (1.50–4.11)	0.0004
H12 lactate > 3 mmol/L	6.70 (3.19–14.1)	<0.0001	1.30 (0.72–2.35)	0.38
H24 lactate > 2.5 mmol/L	5.72 (2.46–13.3)	<0.0001	1.21 (0.63–2.34)	0.57

Results are expressed as odds-ratio and 95%confidence intervals. A *p*<0.05 was considered significant.

As beta-adrenergic agents could influence lactate metabolism, we compared lactate levels in patients receiving or not beta-adrenergic agents (epinephrine, dobutamine) in the first 6 hours after ICU admission. Patients receiving beta-adrenergic agents exhibited higher lactate values on admission (4.95 mmol/L (3.12–9.52) vs. 3.78 mmol/L (1.77–6.80); p = 0.02), at 12 hours (2.50 mmol/L (1.68–6.76) vs. 1.90 mmol/L (1.20–3.10); p<0.01) and 24 hours (2.11 mmol/L (1.23–3.79) vs. 1.40 mmol/L (1.00–2.31); p<0.01).

## Discussion

This study shows an association between lactate levels and outcome in out-of-hospital cardiac arrest patients treated by therapeutic hypothermia. Moreover, increasing values of initial lactate are associated with higher proportions of poor prognosis. To the best of our knowledge, we report the first study including only OHCA patients receiving therapeutic hypothermia. Previous studies reported conflicting results but most of them were conducted before the era of therapeutic hypothermia or included a heterogeneous population of in-hospital and out-of-hospital cardiac arrest patients. These differences might explain partly the disagreements between some of our results and previous publications. Thus, Starodub et al. failed to find an association between initial lactate levels and neurological prognosis but they included a significant proportion of in-hospital cardiac arrest patients (IHCA) [11]. Despite the absence of study comparing specifically IHCA to out-of-hospital counterpart, we presume these 2 populations are different as reported by recent studies [4,20]. In-hospital cardiac arrest patients presented more comorbidity, higher percentages of cardiac origin and shockable rhythm, and a shorter downtime. Most importantly, the cause of death was more frequently due to cardiac abnormalities, whereas the majority of patients suffering OHCA died from post-anoxia encephalopathy [19]. The proportion of patients treated with therapeutic hypothermia represented the major difference between our study and the previous works. Indeed, international guidelines recommend the use of therapeutic hypothermia [17], leading to an increased application of this treatment [4,21–24]. But, as a consequence, this practice modified the prognostic value of different parameters. A longer duration of infusion and a decreased metabolism induced by hypothermia [25] led to the presence of residual sedative drugs, a major confounding factor of early neurological examination. Biomarkers of brain injuries such as S-100B and NSE were considered reliable prognostication tools but therapeutic hypothermia shifted the previously validated thresholds [26]. The influence of therapeutic hypothermia on the prognostic value of lactate could be deduced from the studies performed before and after its implementation. The first 2 studies performed in the 1990’s demonstrated the association of initial lactate with outcome [9,10] whereas the study by Donnino et al. did not [12]. After the implementation of therapeutic hypothermia, studies reported also conflicting results [11,13,14]. All these results suggested an absence or a modest effect of therapeutic hypothermia on prognostic value of initial lactate. Moreover, as the positive studies did not describe a threshold value separating good and poor prognosis, the potential influence of therapeutic hypothermia on lactate levels was difficult to evaluate. Interestingly, initial lactate was not only associated to outcome but also to the type of death, patients dying from MOF presenting higher values than neurologic failure patients. This result is in agreement with a previous study analysing the type of death after OHCA [19].

Besides initial lactate, subsequent measurements were also associated to prognosis, poor outcome patients exhibiting higher values. This result was already demonstrated in previous studies [13,14]. However, multiple conditions can influence lactate levels after resuscitation: cardiogenic shock [27], infusion of adrenaline [28], bowel ischemia [29,30], persistent hypoxia or mitochondrial dysfunction [31]. Thus, subsequent lactate levels do not reflect only ischemia insult, including brain damage.

The initial lactate levels reported in this study are lower in each group compared to most published data [9,10,12–16]. This difference could be explained by several causes such as the site of blood sampling, a different population and the emergency medical system. Thus, venous lactate concentrations are slightly higher than arterial levels [32]. The comparison of the characteristics of the populations across the studies is challenging. Indeed several parameters account for these differences: proportion of IHCA [11], selection of the population [10] and poor outcome ranging from 51% [10] to 75% [15]. Actually, the main factor explaining different lactate levels could be the organisation of care. After successful resuscitation and in absence of other cause of hyperlactatemia, lactate metabolism leads to a rapid decrease of its value. For this reason, the time between sustained return of spontaneous circulation and blood sampling could be a major determinant of lactate levels. We report a median duration of 160 minutes between cardiac arrest and first lactate measurement in ICU. Unfortunately, this parameter was not reported in the different studies. However, a shorter time could be expected in a paramedics staffed emergency system where patients are transported to the emergency department, sometimes during resuscitative efforts. In our work, initial lactate level was measured upon arrival in ICU. But a proportion of our patients were firstly managed in an emergency department or in a cardiology unit to perform coronary angiogram and percutaneous coronary intervention when indicated, leading to variable times in initial arterial lactate measurements. This hypothesis could be supported by the presence of comparable lactate values at 12 and 24 hours in our study compared to others [13,14].

Lactate is a physiological substrate derived from pyruvate reduction during glycolysis. In hypoxemic patients, pyruvate does not enter the Krebs cycle leading to accumulation and consequently hyperlactatemia. This increased serum lactate level correlates with the ischemia duration in a model of cardiac arrest [8]. On the other hand, the duration of no-flow is also correlated to mortality as previously reported [33]. Taken together, these results may explain the relationship that we found between quartiles of initial lactate levels and outcome. Previous studies demonstrated a similar association by using arbitrary ranges of lactate levels instead of quartiles [9,13,15].

Although our study showed an association of lactate levels and quartiles of lactate with prognosis, these parameters alone cannot be used to establish a prognosis after out-of-hospital cardiac arrest. Recent guidelines recommend the use of a multimodal strategy for prognostication [34]. This is emphasized by Oddo et al. who showed that a combination of clinical examination, electroencephalography reactivity and serum NSE provides a good outcome predictive performance in a population of 134 post cardiac arrest patients treated with therapeutic hypothermia [35].

While offering novel insights, some aspects of our study have to be interpreted with caution. The first limitation is the retrospective design of the study. Some interesting parameters such as Glasgow motor score, biochemical markers or EEG were not available for all patients. The second limitation is the time of neurological evaluation performed at ICU discharge and not at 6 or 12 months.

## Conclusions

After out-of-hospital cardiac arrest patients treated with therapeutic hypothermia, lactate concentrations during the first 24 hours seem associated to outcome. Increasing values of lactate are correlated with a poor prognosis. Patients dying from multiorgan failure seem to exhibit higher initial lactate concentrations than patients succumbing from neurological failure. However, in the light of guidelines, it is difficult to consider it alone for prognostication of neurological outcome. Further prospective studies are needed to evaluate lactate associated to a bundle of parameters.
